# Anti-IL-20 Monoclonal Antibody Suppresses Prostate Cancer Growth and Bone Osteolysis in Murine Models

**DOI:** 10.1371/journal.pone.0139871

**Published:** 2015-10-06

**Authors:** Yu-Hsiang Hsu, Cheng-Ying Wu, Chung-Hsi Hsing, Wei-Ting Lai, Li-Wha Wu, Ming-Shi Chang

**Affiliations:** 1 Institute of Clinical Medicine, College of Medicine, National Cheng Kung University, Tainan, Taiwan; 2 Department of Biochemistry and Molecular Biology, College of Medicine, National Cheng Kung University, Tainan, Taiwan; 3 Department of Anesthesiology, Chi-Mei Medical Center, Tainan, Taiwan; 4 Institute of Molecular Medicine, College of Medicine, National Cheng Kung University, Tainan, Taiwan; 5 Department of Anesthesiology, College of Medicine, Taipei Medical University, Taipei, Taiwan; Faculté de médecine de Nantes, FRANCE

## Abstract

Interleukin (IL)-20 is a proinflammatory cytokine in the IL–10 family. IL–20 is associated with tumor promotion in the pathogenesis of oral, bladder, and breast cancer. However, little is known about the role of IL–20 in prostate cancer. We hypothesize that IL–20 promotes the growth of prostate cancer cells. Immunohistochemical staining showed that IL–20 and its receptors were expressed in human PC–3 and LNCaP prostate cancer cell lines and in prostate tumor tissue from 40 patients. *In vitro*, IL–20 upregulated N-cadherin, STAT3, vimentin, fibronectin, RANKL, cathepsin G, and cathepsin K, and increased the migration and colony formation of prostate cancer cells via activated p38, ERK1/2, AKT, and NF-κB signals in PC–3 cells. We investigated the effects of anti-IL–20 monoclonal antibody 7E on prostate tumor growth *in vivo* using SCID mouse subcutaneous and intratibial xenograft tumor models. *In vivo*, 7E reduced tumor growth, suppressed tumor-mediated osteolysis, and protected bone mineral density after intratibial injection of prostate cancer cells. We conclude that IL–20 is involved in the cell migration, colony formation, and tumor-induced osteolysis of prostate cancer. Therefore, IL–20 might be a novel target for treating prostate cancer.

## Introduction

Prostate cancer is the second most incident cancer and the sixth cause of cancer death in men worldwide [1]. The majority of men with advanced prostate cancer have sclerotic bone metastases, which cause severe pain and pathologic bone fractures [2, 3]. Invasion of the bone compartment by cancer cells causes an imbalance in osteoclast and osteoblast activity that in turn interrupts bone homeostasis [4]. These cancer-induced bone responses favor the survival and growth of cancer cells in their new environment. Although prostate cancer bone metastases are primarily osteoblastic in nature, there is increasing evidence that osteoclast-mediated osteolysis also contributes to bone morbidity in prostate cancer patients with bone metastases [5, 6].

Others report that breast and prostate cancers induce osteoclast activation by releasing soluble factors such as IL–1, IL–6, and macrophage colony-stimulating factor (M-CSF). Most of these mediators act on osteoblasts and stromal cells, and contribute to osteolytic lesions by upregulating receptor activator of NF-κB ligand (RANKL), which provides one possible mechanism to explain the increased bone resorption in bone metastasis [6, 7]. RANKL binds to its receptor (RANK) on the cell membrane of osteoclast, which leads to the differentiation and maturation of the osteoclasts [8]. Cathepsin K, a cysteine protease secreted by osteoclasts and prostate cancer cells, degrades extracellular matrix during bone resorption [9]. Cathepsin G, a chemoattractant for osteoclast precursors, is capable of processing RANKL to a soluble form (sRANKL) that promotes osteoclast activation [10, 11]. Blocking cathepsin G and cathepsin K significantly reduces tumor-induced osteolysis, which suggests that cathepsin K and cathepsin G are crucial in the microenvironment of cancer-induced osteolytic lesions [10, 12].

Inflammation is critically related to tumor progression. It affects tumorigenesis at the molecular level by modulating the tumor microenvironment and regulating the balance of cytokines, chemokines and transcriptional factors [13, 14]. The epithelial-mesenchymal transition (EMT) is critical for cancer metastasis. EMT changes the behavior of cancer cells and causes them to invade the surrounding stroma, leads to their intravasation, dissemination, and colonization of distant sites [15, 16]. During EMT, tumor cells, which are epithelial-like cells, acquire mesenchymal characteristics, and the loss of the epithelial marker E-cadherin leads to an increase in the mesenchymal markers N-cadherin, fibronectin, and vimentin [17]. Several EMT inducers, such as Snail and Twist, are transcription factors that repress E-cadherin expression [16, 18]. Other studies [19, 20] have reported that cancer cells promoted EMT by activating the STAT3 signaling pathway.

IL–20 is a member of the IL–10 family, which includes IL–10, IL–19, IL–20, IL–22, IL–24, and IL–26 [21, 22]. It signals through two types of heterodimer receptor complex: IL-20R1/IL-20R2 and IL-22R1/IL-20R2 [23]. IL–20 is involved in several inflammatory diseases, such as rheumatoid arthritis [24], atherosclerosis [25], psoriasis [26, 27], osteoporosis [28], oral cancer [29], and breast cancer [30].

Prostate cancer is a complex disease in which metastasis to the bone is a cause of morbidity and may precede metastasis to other vital organs. We previously [28, 30] showed that IL–20 not only promotes breast tumor proliferation and migration, but also modulates osteoclast differentiation by upregulating RANKL and RANK. Anti-IL–20 monoclonal antibody (mAb) 7E decreased osteolytic bone lesions in mouse models of breast cancer and protected ovariectomized mice against osteoporotic bone loss, both of which support the notion that IL–20 is critical for regulating of tumor-mediated osteolysis. We hypothesize that IL–20 promotes the growth of prostate cancer cells. Therefore, we investigated the expression of IL–20 and its biological function in prostate cancer cells and assessed the therapeutic potential of 7E in xenograft prostate cancer mouse models.

## Materials and Methods

### Immunohistochemistry

Paraffin-embedded tissue sections of 40 human prostate cancer specimens were obtained from a commercial prostate cancer tissue array (SuperBioChips Laboratories, Seoul, Korea), and were used for immunohistochemical (IHC) staining with anti-IL–20 (7E), anti-IL-20R1, anti-IL-20R2, or anti-IL-22R1 mAb (R&D Systems, Minneapolis, MN) as previously described [31]. Incubating paraffin tissue sections with mouse IgG1 isotype (clone 11711; R&D Systems) instead of primary antibody was the negative control. We used 3 μg/ml as the working concentration for each primary antibody and for the control mouse IgG1. Two pathologists trained in prostate pathology and blinded to the sample sources analyzed the histology and the expression levels of IL–20 and its receptors from each patient. The scoring of immunohistochemical stains in each specimen was determined using a histological score (H) [32] that was calculated with the following equation: H = ΣPi (i + 1), where i is the staining intensity of the stained tumor cells (0–4+), and Pi is the percentage (range: 0–100%) of stained tumor cells for each intensity. The IL–20, IL-20R1, IL-20R2, and IL-22R1 immunostaining were labeled low-expression (H < 200) or high-expression (H ≥ 200). Immunocytochemical staining of IL–20 and its receptors in PC–3 cells was done using the same protocol as described above.

### Cell culture

Prostate cancer cell lines were purchased from the American Type Culture Collection (Manassas, VA). Human prostate cancer cell lines, PC–3 and LNCaP, were maintained in RPMI–1640 with 10% fetal bovine serum (FBS) (Life Technologies, Rockville, MD), 100 μg/ml of streptomycin, and 100 U/ml of penicillin. Cells were incubated at 37°C in a humidified atmosphere containing 5% CO_2_.

### Cell proliferation assay

PC–3 cells (3×10^4^) were cultured overnight and then exposed to human (h) IL–20 (200 ng/ml) for 72 hours in medium containing 1% FBS. To confirm the specific activity of IL–20, 7E (2 μg/ml) was added to the culture system, either alone or together with IL–20 at a 10:1 (7E: IL–20) concentration ratio. The cells were then incubated with 1 mg/ml of methylthiazol tetrazolium (MTT) (Sigma-Aldrich) for 3 hours and the MTT-formazan crystals were dissolved in dimethylsulfoxide (DMSO) (Sigma-Aldrich). Absorbance was measured at 550 nm.

### Cell migration assay

Cell migration was assayed using a modified Boyden chamber with a polycarbonate filter with 8-μm pores (Nucleopore, Cabin John, MD). The upper wells were loaded with PC–3 cells (5×10^4^). The lower chambers were filled with hIL–20 (200 ng/ml), 7E (2 μg/ml), mIgG (2 μg/ml), hIL–20 plus 7E, or hIL–20 plus mIgG in RPMI–1640 containing 1% FBS. RPMI–1640 with 1% FBS was used as a negative control. The cells were allowed to migrate for 8 hours, and then they were stained and counted. The experiment was done for three times using quadruplicate wells.

### Real-time migration assay

PC–3 cells were seeded at 5×10^4^ cells/ml in 6-well dishes and allowed to attach for 18 hours. The cells were then exposed to medium containing hIL–20 (200 ng/ml), 7E (2 μg/ml), mIgG (2 μg/ml), hIL–20 plus 7E, or hIL–20 plus mIgG. Cell migration kinetics was recorded using a fluorescent cell analyzer (JuLI Smart; Montreal Biotechnologies Inc. (MBI), Dorval, Montreal, Canada) for 18 hours and then analyzed using ImageJ Software (http://rsbweb.nih.gov/ij/).

### Soft agar colony-forming assay

Cells exhibiting exponential growth were suspended in complete growth medium containing 0.35% Bacto-agar (A–6013 Type 1 Low EEO; Sigma-Aldrich) and overlaid on 0.5% agarose gel in 30-mm dishes (1×10^4^ cells/dish). Medium containing IL–20 (200 ng/mL), 7E (2 μg/ml), mIgG (2 μg/ml), hIL–20 plus 7E, or hIL–20 plus mIgG was overlaid on the top agar. The dishes were maintained at 37°C in a humidified incubator (5% CO_2_, 95% O_2_) for three weeks. During this period, the medium was changed every 2 days. The number of visible colonies (>50 μm) was counted under a microscope. All experiments were done in triplicate.

### Real-time quantitative polymerase chain reaction (RT-qPCR)

To analyze the expression of IL–20 and its receptors, total RNA from PC–3 and LNCaP cells was extracted using a reagent (Trizol; Invitrogen, Carlsbad, CA), and then total RNA underwent reverse transcription (Clontech, Palo Alto, CA) according to the manufacturer’s instructions. The amplified template was detected using SYBR Green with a real-time PCR system (StepOnePlus; Applied Biosystems) with gene-specific primers. Glyceraldehyde phosphate dehydrogenase (GAPDH) was used as an internal control. To examine the expression of E-cadherin, N-cadherin, STAT3, vimentin, fibronectin, RANKL, cathepsin G, and cathepsin K, PC–3 cells were incubated with hIL–20 (200 ng/ml) for 2 to 6 hours. The expression levels of these genes were analyzed using SYBR Green (Applied Biosystems) as the interaction agent. Quantification analysis of mRNA was normalized with human GAPDH as the housekeeping gene. Relative multiples of change in mRNA expression was determined by calculating 2^−ΔΔCt^.

### Western blotting

PC–3 cells (2×10^5^) were stimulated with hIL–20 (200 ng/ml) (R&D Systems) for the indicated times. Total cell lysate was collect by adding 1× RIPA buffer containing phenylmethanesulfonyl fluoride (PSMF) (RIPA:PSMF = 10:1) and centrifuged at 13000 rpm at 4°C to collect the supernatant. Western blotting with antibody specific for phosphor-p38, phosphor-extracellular signal-regulated kinase (ERK1/2), phosphor-AKT, and phosphor-nuclear factor-kappa B (NF-κB) (Cell Signaling Technology) was used following the manufacturer’s instructions. β-actin was used as an internal control (Cell Signaling Technology). Quantification of proteins in western bands was done using ImageJ software.

### Enzyme-linked immunosorbent assay (ELISA)

PC–3 cells were incubated with hIL–20 (100, 200, or 400 ng/ml) and the cultured media were collected and determined using an sRANKL ELISA kit (Peprotech, Rocky Hill, NJ) according to the manufacturer’s instructions. Cathepsin G-specific inhibitor, N-tosyl-l-phenylalanine chloromethyl ketone (TPCK; Sigma-Aldrich) was used to block the protease activity of cathepsin G. PC–3 cells were preincubated with 1 μM of TPCK for 1 hour and then treated with hIL–20 for another 72 hours. TPCK was dissolved in 100% ethanol (EtOH), and then diluted in culture medium. The vehicle control was 0.0175% EtOH in culture medium. The level of sRANKL in the final cultured medium was analyzed by ELISA.

### Prostate cancer PC-3-bearing tumor model

All animal experiments were conducted according to the protocols based on the Taiwan National Institutes of Health (Taipei, Taiwan) standards and guidelines for the care and use of experimental animals. The research procedures were approved by the Animal Ethics Committee of National Cheng Kung University. All efforts were made to minimize animal suffering and to reduce the number of animals used. Eight-week-old male severe combined immunodeficient (SCID) mice were used in all experiments. Briefly, mice were anesthetized using an intraperitoneal (i.p.) injection of pentobarbital (50 mg/kg) and were then injected with buprenorphine (2 mg/kg; i.p.) for surgical analgesia. The left mammary fat pad of each mouse was subcutaneously injected with PC–3 cells (1×10^6^). The success rate for subcutaneous (s.c.) tumor implantation was 100%. The mice were then randomly assigned to 3 groups (n = 4 in each group), and treated with phosphate-buffered saline (PBS), 7E (10 mg/kg; s.c.), or mouse immunoglobulin G (mIgG; 10 mg/kg; s.c.) three times per week for the duration of the treatment regimen. The antibody was injected (s.c.) into the periphery of the growing tumor in tumor-bearing SCID mice. Healthy controls were not injected with tumor cells. The tumor size was measured with a caliper in three perpendicular dimensions and calculated using the following formula: tumor size = 0.5 × (length × width × depth). Forty days after the tumor cells had been injected, the mice were killed using CO_2_, and the tumor tissue was harvested and weighed. To analyze the expression levels of IL–20 and cathepsin G, the tumor tissue isolated from the 4 mice in each group was collected, and the total RNA was extracted for further analysis.

### Intratibial injection of PC–3 in SCID mice

Eight-week-old male SCID mice were anesthetized using an injection of pentobarbital (50 mg/kg) and then of buprenorphine (2 mg/kg; i.p.) for surgical analgesia. Each was given a medial parapatellar incision and a needle was placed in the intramedullary canal of the tibia. PC–3 cells, at a concentration of 2×10^5^/100 μl, were slowly injected into the tibia and the incision was closed with 5–0 chromic sutures. For postoperative analgesia, buprenorphine (2 mg/kg; i.p.) was injected once daily for 3 days post-surgery. The success rate for intratibial tumor implantation was 100%. The mice were then randomly assigned to three groups (n = 5 in each group) and injected with vehicle (PBS; i.p.), mIgG (10 mg/kg; i.p.), or 7E (10 mg/kg; i.p.) three times per week. Seven weeks after treatments began, the mice were killed using CO_2_, and their tibial metaphyses were analyzed *in vivo* using micro-computed tomography (micro-CT) with a high-resolution, low-dose X-ray scanner. Bone mineral density (BMD) was analyzed in 50 consecutive slices. The results were calculated as a percentage versus values from a healthy control.

### Statistical analysis

Prism 5.0 (GraphPad Software, San Diego, CA) was used for the statistical analysis. A one-way analysis of variance (ANOVA) nonparametric Kruskal-Wallis test was used to compare the data between groups. Post hoc comparisons were done using Dunn's multiple comparison test. Data are means ± standard deviation (SD). Significance was set at *P* < 0.05.

## Results

### Expression of IL–20 and its receptors in patients with prostate cancer

**Fig 1:**
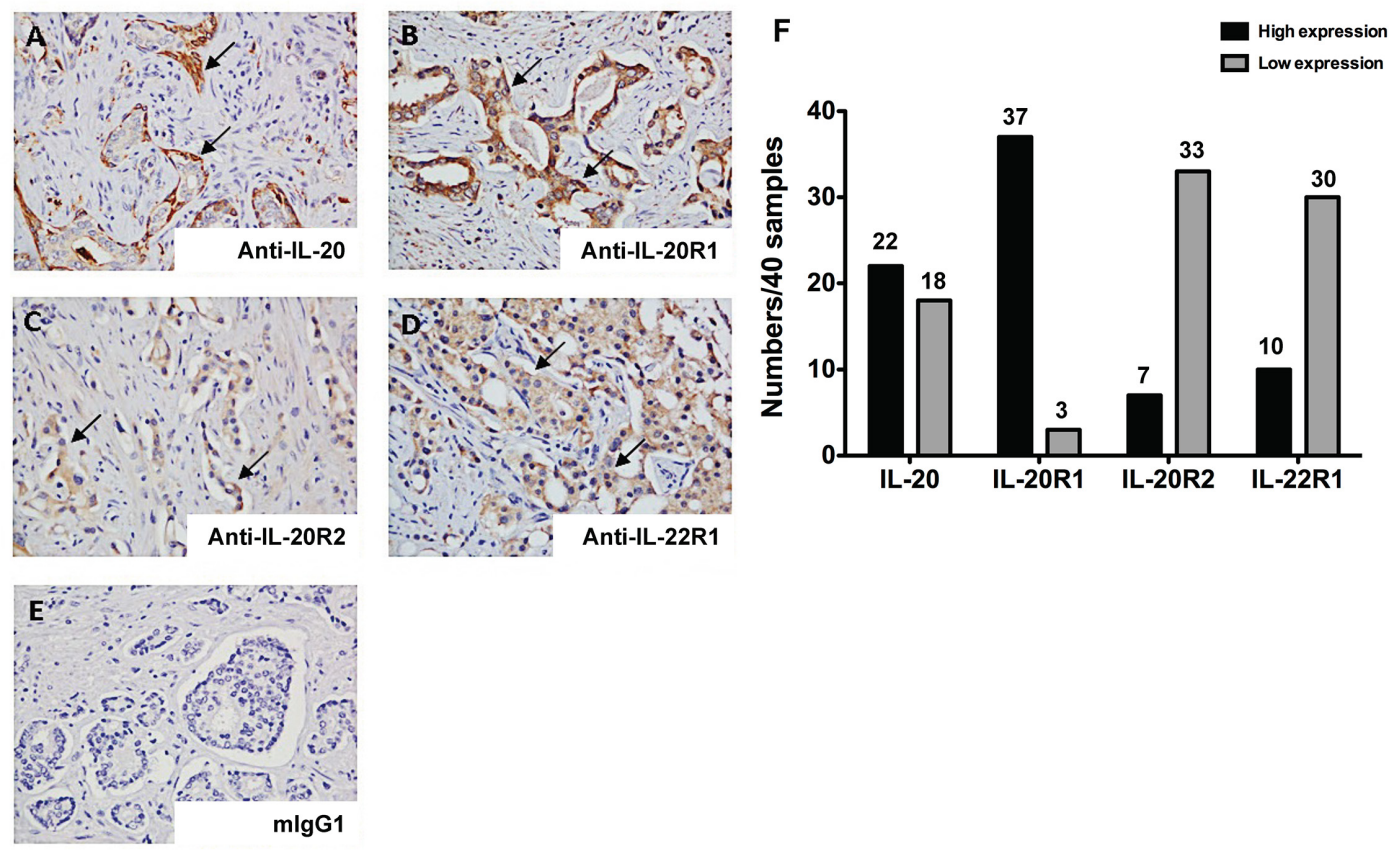
Expression of IL–20 and its receptors in prostate cancer. (A-E) Surgically biopsied prostate adenocarcinoma tissue samples (stage II, n = 8; stage III, n = 32) from 40 patients were obtained from a commercial prostate cancer tissue array. IHC staining with anti-IL–20, anti-IL-20R1, anti-IL-20R2, and anti-IL-22R1 mAbs showed that IL–20 and its receptors (IL-20R1, IL-20R2, and IL-22R1) were stained. Mouse IgG1 (mIgG1) isotype was the negative control. Arrows indicate prostate cancer cells. Magnification: 200×. Data are representative of 2 independent experiments with similar results. (F) Quantitation of staining intensity of anti-IL–20, anti-IL-20R1, anti-IL-20R2, and anti-IL-22R1 mAbs from 40 human prostate cancer specimens. IHC, immunohistochemical staining; mAb, monoclonal antibody; mIgG, mouse immunoglobin.

Forty prostate adenocarcinoma tissue samples (stage II, n = 8; stage III, n = 32) were IHC stained with anti-IL–20 mAbs. Staining intensity was high-expression in 22 samples (Fig 1A) and low-expression in 18 samples. To investigate whether prostate cancer cell is the target cell for IL–20, we used IHC staining to analyze the expression levels of IL–20’s receptors (IL-20R1, IL-20R2, and IL-22R1) in prostate adenocarcinoma tissue samples from 40 patients. The prostate carcinoma cells were all positively stained with anti-IL-20R1, anti-IL-20R2, and anti-IL-22R1 mAbs (Fig 1B, 1C and 1D). The intensity of the IHC staining of prostate carcinoma tissues was heterogeneous (Fig 1F). Anti-IL–20 and anti-IL-20R1 mAbs are highly stained on tumor cells, but anti-IL-20R2 and anti-IL-22R1 mAbs are not (Fig 1A, 1B, 1C and 1D, arrows) on the representative carcinoma tissues. The expression of IL-20R1, IL-20R2, and IL-22R1 was high in 37, 7, and 10 samples, respectively.

### Cell proliferation was inhibited in 7E-treated PC–3 cells

**Fig 2:**
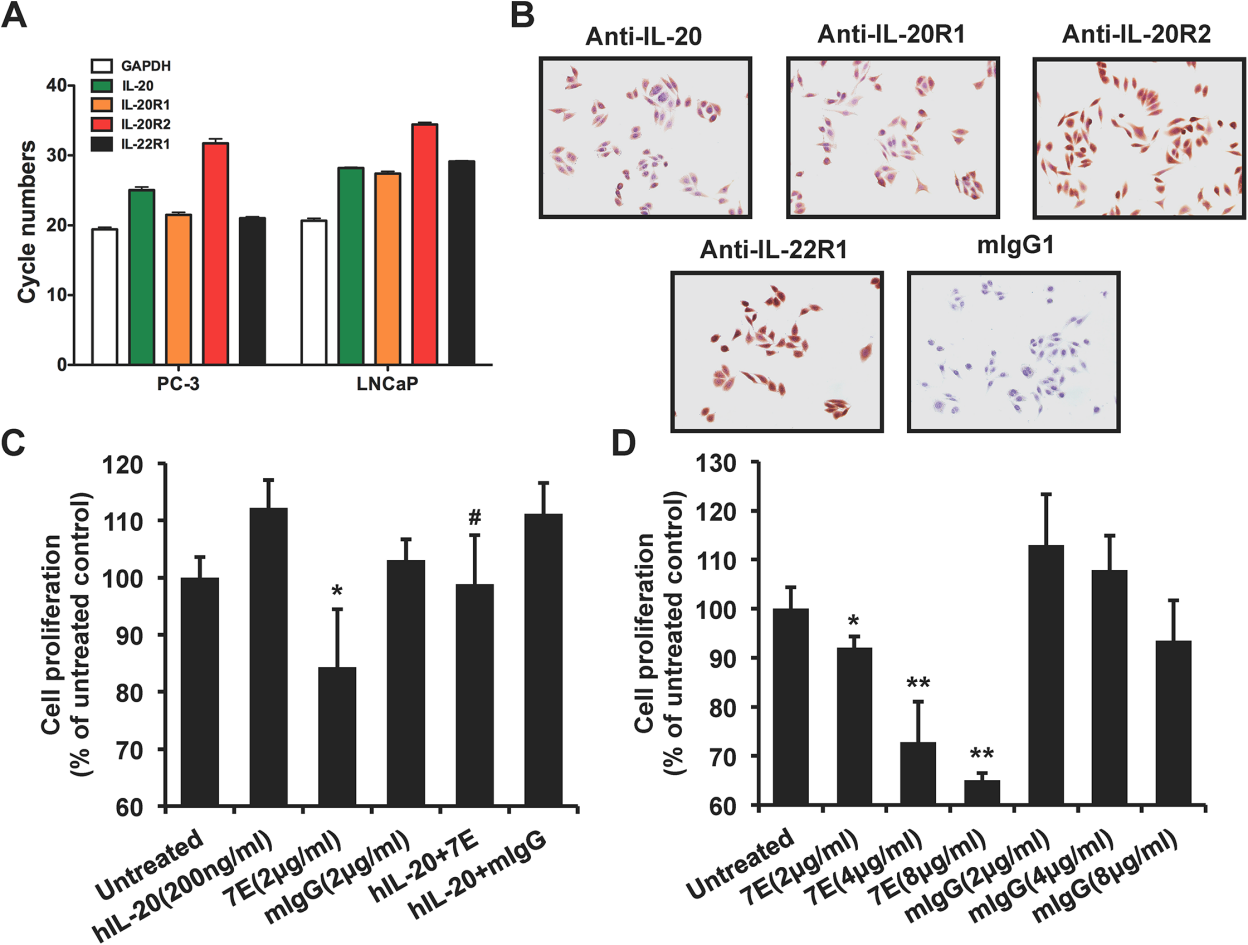
Anti-IL–20 mAb inhibited cell proliferation in PC–3 cells. (A) RT-qPCR showed that IL–20 and its receptors were expressed in prostate cancer PC–3 and LNCaP cells. Data are representative of 2 independent experiments with similar results. (B) IHC showed that IL–20 and its receptors were expressed in prostate cancer PC–3 cells. Data are representative of 2 independent experiments with similar results. (C) An MTT assay showed that cell proliferation was inhibited in 7E-treated PC–3 cells. Medium alone was used as a negative control. 7E was used to inhibit the activity of hIL–20. *p < 0.05 versus untreated controls, #p < 0.05 versus the hIL–20 group. Data are the means ± SD of three independent experiments. (D) An MTT assay showed that cell proliferation was dose-dependently inhibited in 7E-treated PC–3 cells. *p < 0.05, **p < 0.01 versus mIgG controls. Data are the means ± SD of three independent experiments. RT-qPCR, real-time quantitative polymerase chain reaction; MTT, methylthiazol tetrazolium; 7E, anti-IL–20 monoclonal antibody; hIL–20, human interleukin–20.

**Fig 3:**
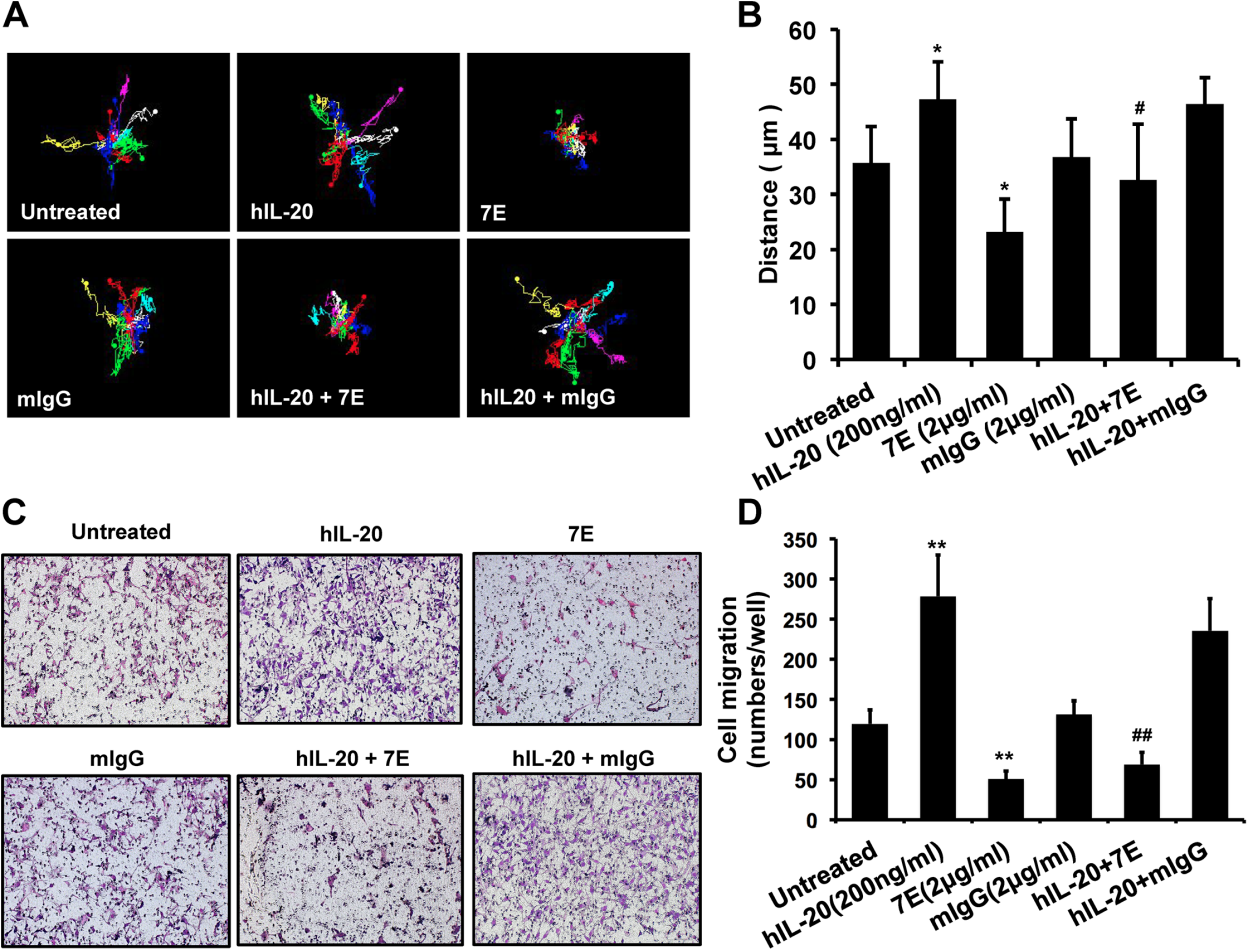
Cell migration was promoted in IL-20-treated PC–3 cells. (A-B) Cell migration was evaluated using real-time migration assay. PC–3 cells were incubated with medium containing hIL–20 (200 ng/ml), 7E (2 μg/ml), mIgG (2 μg/ml), hIL–20 plus 7E, or hIL–20 plus mIgG. Cell migration kinetics was recorded using a smart fluorescent cell analyzer for 18 hours. (A) Representative time-lapse images for tracking motion distance of PC–3 cells are shown for each group. The motion distance of each cell is presented in different colors (count = 7 cells). (B) Quantization of the motion distance (in μm) of PC–3 cells (count = 7 cells). Mouse IgG was the negative control of 7E. *p < 0.05 versus untreated controls, #p < 0.05 versus the hIL–20 group. Data are the means ± SD of three independent experiments, each done in quadruplicate. (C-D) Cell migration was also evaluated using a modified Boyden chamber assay. The upper wells were loaded with 5×10^4^ PC–3 cells. The lower chambers were filled with hIL–20 (200 ng/ml), 7E (2 μg/ml), mIgG (2 μg/ml), hIL–20 plus 7E, or hIL–20 plus mIgG in RPMI–1640 containing 1% FBS. RPMI–1640 with 1% FBS was used as a negative control. The cells were allowed to migrate for 8 hours. (C) Representative Giemsa staining photos are shown for each group. (D) Quantitation of migrated cells per well. **p < 0.01 versus untreated controls. ##p < 0.01 versus the hIL–20 group. Data are the means ± SD of three independent experiments, each done in quadruplicate.

To clarify the role of IL–20 in the pathogenesis of prostate cancer, we first examined whether IL–20 and its receptors (IL-20R1, IL-20R2, and IL-22R1) were expressed in prostate cancer cell lines. RT-qPCR and IHC staining showed that IL–20 and its receptors were all expressed in PC–3 cells (Fig 2A and 2B), and in LNCaP cells (Fig 2A). The first step in tumor progression is thought to be the result of a genetic alteration that leads to the abnormal proliferation of a single cell. To determine whether IL–20 promoted PC–3 cell proliferation, we used an MTT assay, which showed that IL–20 did not significantly promote cell proliferation of PC–3 cells, but that cell proliferation was dose-dependently inhibited in 7E-treated PC–3 cells (Fig 2C and 2D). Tumor progression involved cell migration and metastasis to distant organs. A real-time migration assay showed that cell migration was increased in IL-20-treated PC–3 cells compared with untreated controls, the activity of which was attenuated by 7E (Fig 3A and 3B). Moreover, a Boyden chamber assay showed similar results (Fig 3C and 3D).

### Colony formation was promoted in IL-20-treated PC–3 cells

**Fig 4:**
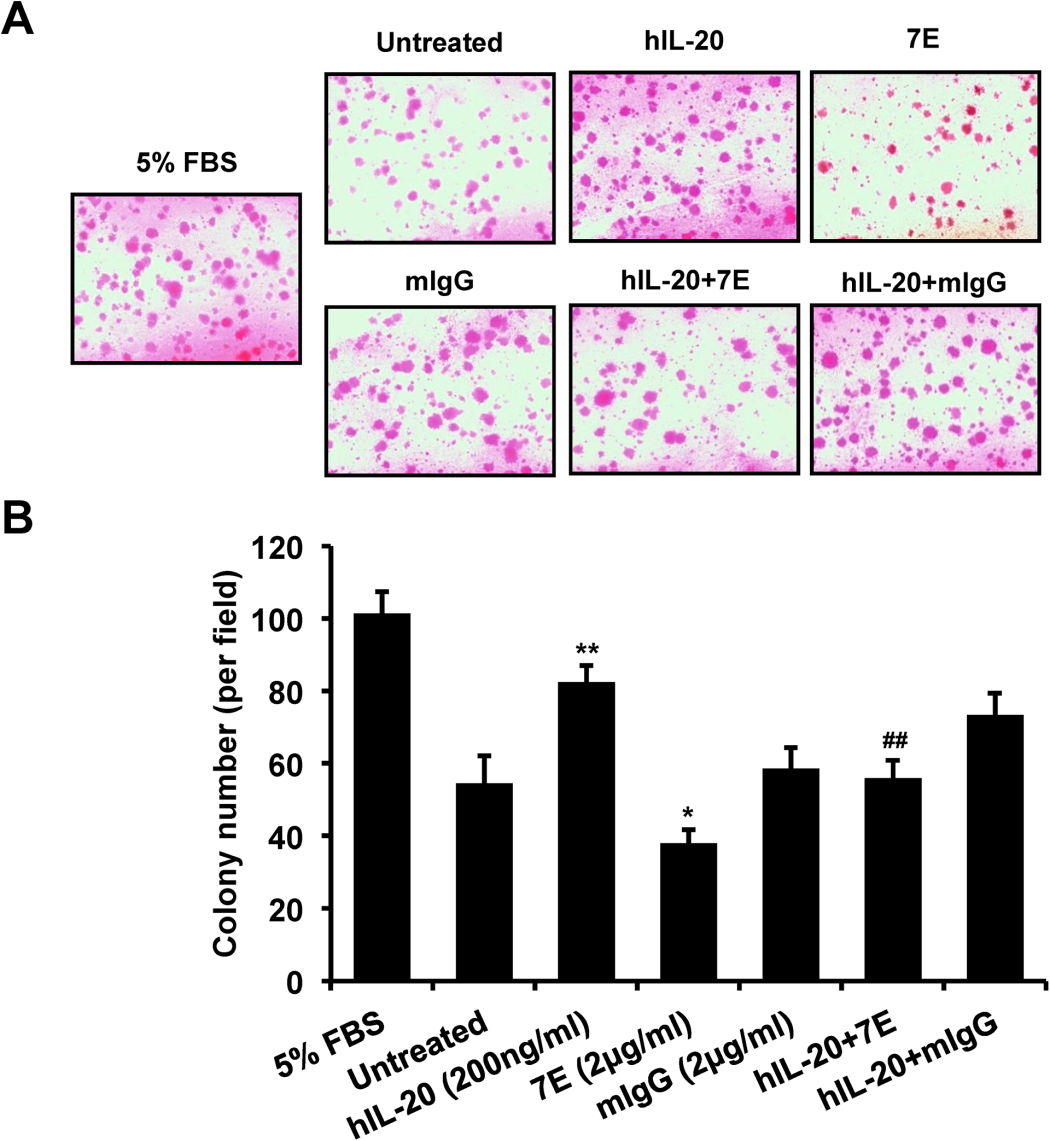
Colony formation was promoted in IL-20-treated PC–3 cells. PC–3 cells (1×10^4^/well) were incubated with hIL–20 (200 ng/ml), 7E (2 μg/ml), mIgG (2 μg/ml), hIL–20 plus 7E, or hIL–20 plus mIgG for 3 weeks. The culture medium was changed every 2 days. (A) Representative photos are shown for each group. (B) Colony formation was significantly higher in IL-20-treated PC–3 cells. 7E (2 μg/ml) was used to inhibit the activity of IL–20. *p < 0.05, **p < 0.01 versus untreated controls, ##p < 0.01 versus the hIL–20 group. Values are the means ± SD of three independent experiments, each done in triplicate.

The initial step of the local invasion of prostate cancer is an increase in the colony formation of the cancer cells. A soft agar colony formation assay showed that anchorage-independent colony formation was significantly higher in IL-20-treated PC–3 cells than in untreated controls, the activity of which was attenuated by 7E (Fig 4A and 4B).

### Signal transduction was induced in IL-20-treated PC–3 cells

**Fig 5:**
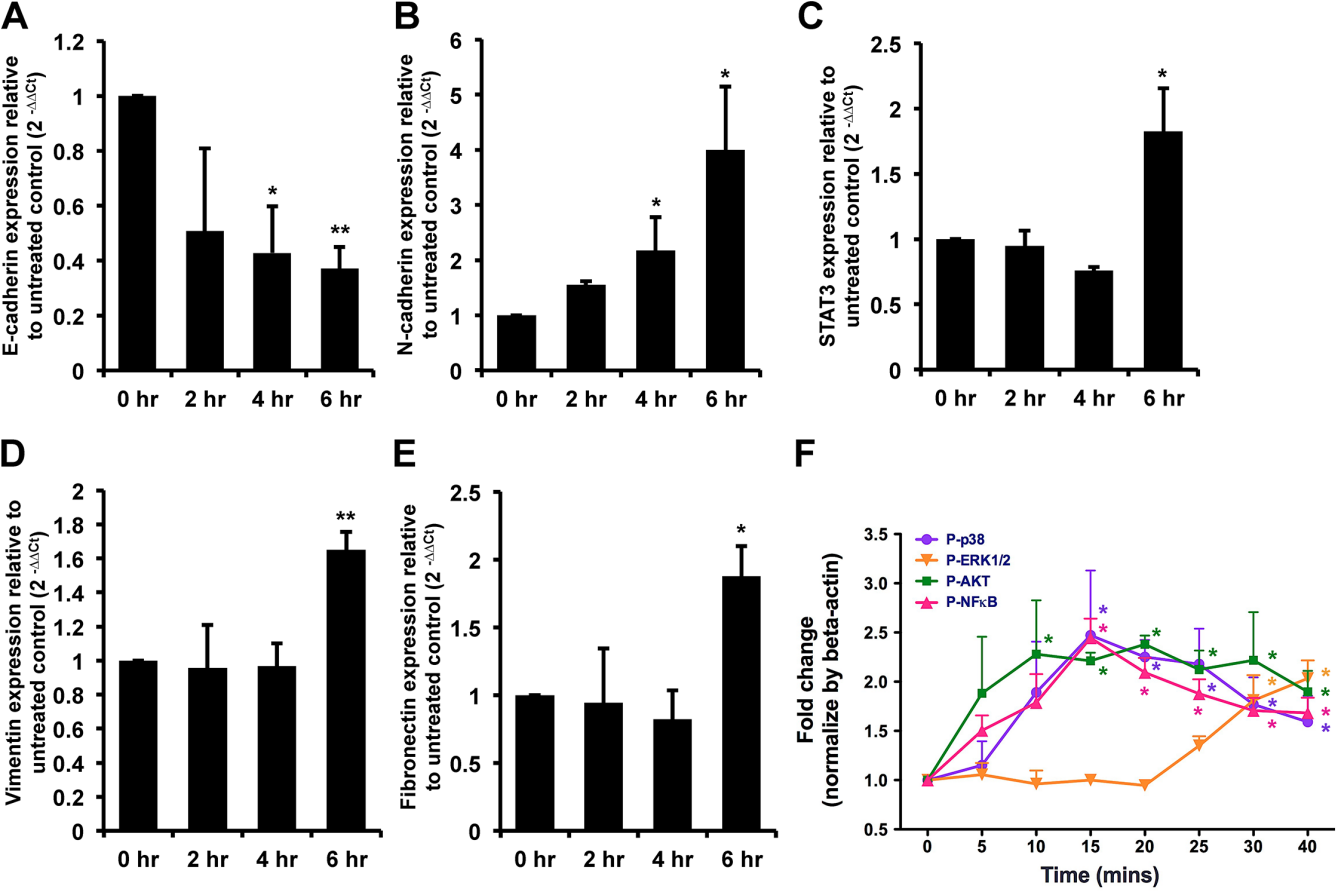
N-cadherin, STAT3, vimentin, fibronectin, and activated intracellular signaling were upregulated in IL-20-treated PC–3 cells. (A-E) PC–3 cells were treated with hIL–20 (200 ng/ml) for the indicated times, and the expression levels of E-cadherin, N-cadherin, STAT3, vimentin, and fibronectin were analyzed using RT-qPCR with specific primers. *p < 0.05, **p < 0.01 versus 0-hour controls. Data are representative of 3 independent experiments, each done in triplicate. (F) PC–3 cells (2×10^5^) were incubated with hIL–20 (200 ng/ml) for the indicated time periods, and then cell lysates were collected and analyzed using immunoblotting with specific antibodies against phospho-p38, phosphor-ERK1/2, phosphor-AKT, and phosphor-NF-κB. β-actin antibody was used as an internal control. Quantification of bands was done using ImageJ software. *p < 0.05 versus 0-min controls. Data are the means ± SD of three independent experiments.

Epithelial-mesenchymal transition (EMT) is fundamental in tumor progression and metastasis. To investigate whether IL–20 is involved in prostate tumor metastasis through EMT, RT-qPCR was used to analyze the expression of the epithelial marker E-cadherin, N-cadherin, STAT3, vimentin, and the mesenchymal marker fibronectin in PC–3 cells incubated with IL–20. It showed that E-cadherin had been downregulated (Fig 5A), and N-cadherin, STAT3, vimentin, and fibronectin had been significantly upregulated (Fig 5B, 5C, 5D and 5E), while in 7E-treated cells, this upregulation was attenuated. To clarify the possible mechanism between IL–20 and tumor progression, the signal molecules of ERK1/2, AKT, NF-κB, and p38 were assessed and found to be phosphorylated in IL-20-treated PC–3 cells (Fig 5F).

### RANKL, cathepsin G, and cathepsin K transcripts and sRANKL protein production were induced in IL-20-treated PC–3 cells

**Fig 6:**
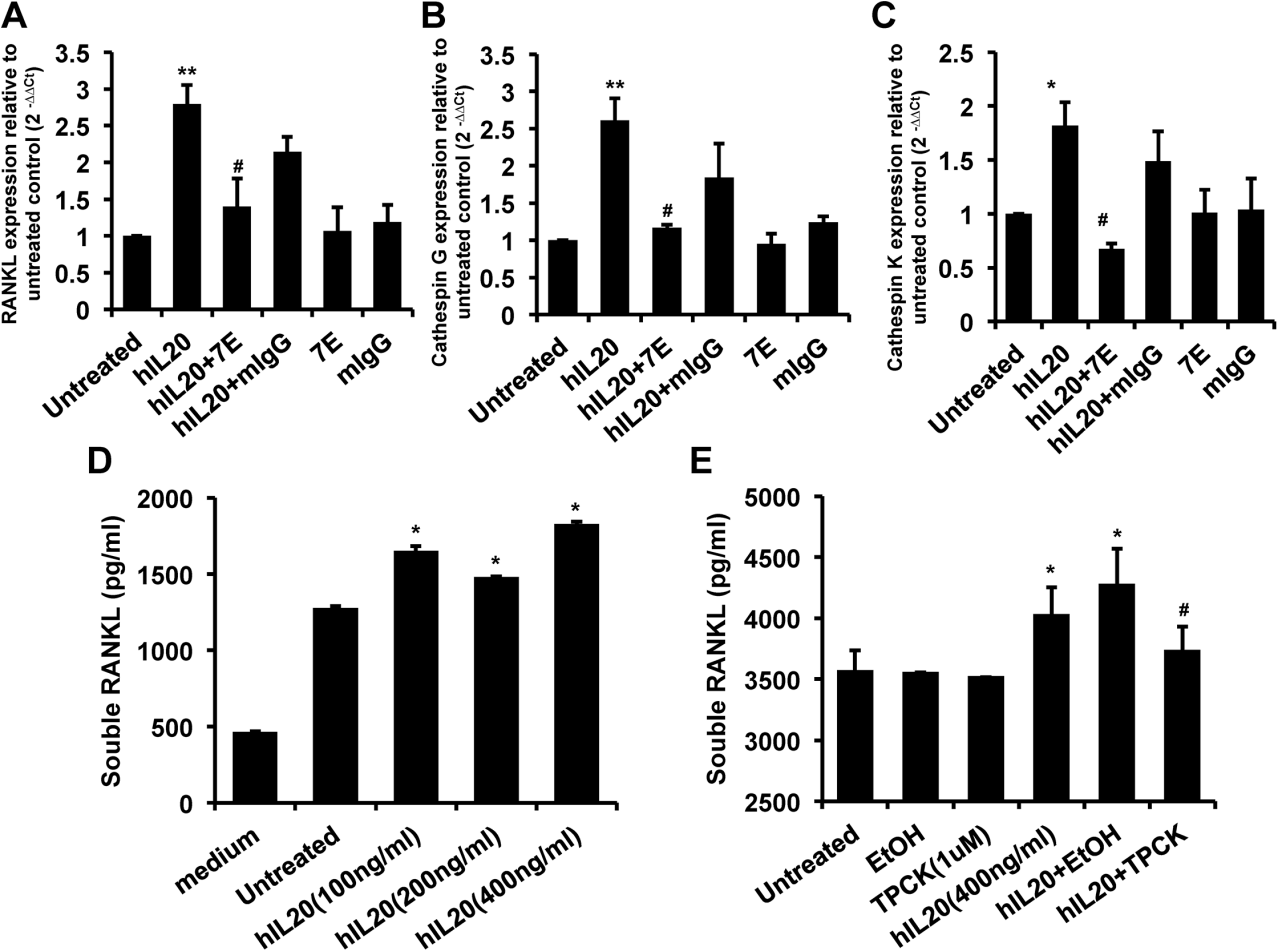
RANKL, cathepsin G, and cathepsin K transcripts were upregulated and sRANKL protein production was promoted in IL-20-treated PC–3 cells. (A-C) PC–3 cells were treated with hIL–20 (200 ng/ml), 7E (2 μg/ml), mIgG (2 μg/ml), hIL–20 plus 7E, or hIL–20 plus mIgG for 6 hours, and the expression levels of RANKL, cathepsin G, and cathepsin K were analyzed using RT-qPCR with specific primers. *p < 0.05, **p < 0.01 versus untreated control, #p < 0.05 versus the hIL–20 group. Data are representative of 3 independent experiments, each done in triplicate. (D) PC–3 cells were incubated with hIL–20 (100, 200, or 400 ng/ml) for 48 hours, the culture medium was collected and then the concentration of sRANKL was determined using ELISA. *p < 0.05 versus untreated controls. Data are representative of 3 independent experiments, each done in triplicate. (E) PC–3 cells were preincubated with 1 μM of cathepsin G-specific inhibitor (TPCK) for 1 hour and then treated with hIL–20 (400 ng/ml) for 72 hours. The vehicle control was 0.0175% EtOH in culture medium. The concentration of sRANKL was determined using ELISA. *p < 0.05 versus untreated controls, #p < 0.05 versus the hIL–20 plus EtOH vehicle controls. Data are the means ± SD of three independent experiments, each done in triplicate. RT-qPCR, real-time quantitative polymerase chain reaction; sRANKL, soluble RANKL; EtOH, ethanol.

To test whether IL–20 regulates cathepsins and RANKL in prostate cancer, PC–3 cells were treated with IL–20 for 6 hours. An RT-qPCR gene transcript analysis showed upregulated RANKL, cathepsin G and cathepsin K expression in IL-20-treated PC–3 cells, the activity of which was neutralized by 7E (Fig 6A, 6B and 6C). Moreover, an ELISA assay showed a significant (p < 0.05) increase in sRANKL expression in IL-20-treated PC–3 cells (Fig 6D). Therefore, we hypothesized that IL-20-treated PC–3 cells produce cathepsin G and subsequently cleave RANKL to generate sRANKL, which further promotes osteoclast activation in bone microenvironment. To confirm that the cleavage of RANKL was cathepsin G-dependent, we used a specific cathepsin G inhibitor (TPCK, 1 μM) to block the protease activity of cathepsin G. The result confirmed our hypothesis (Fig 6E).

### Tumor growth was inhibited in 7E-treated PC–3 prostate cancer xenografts

**Fig 7:**
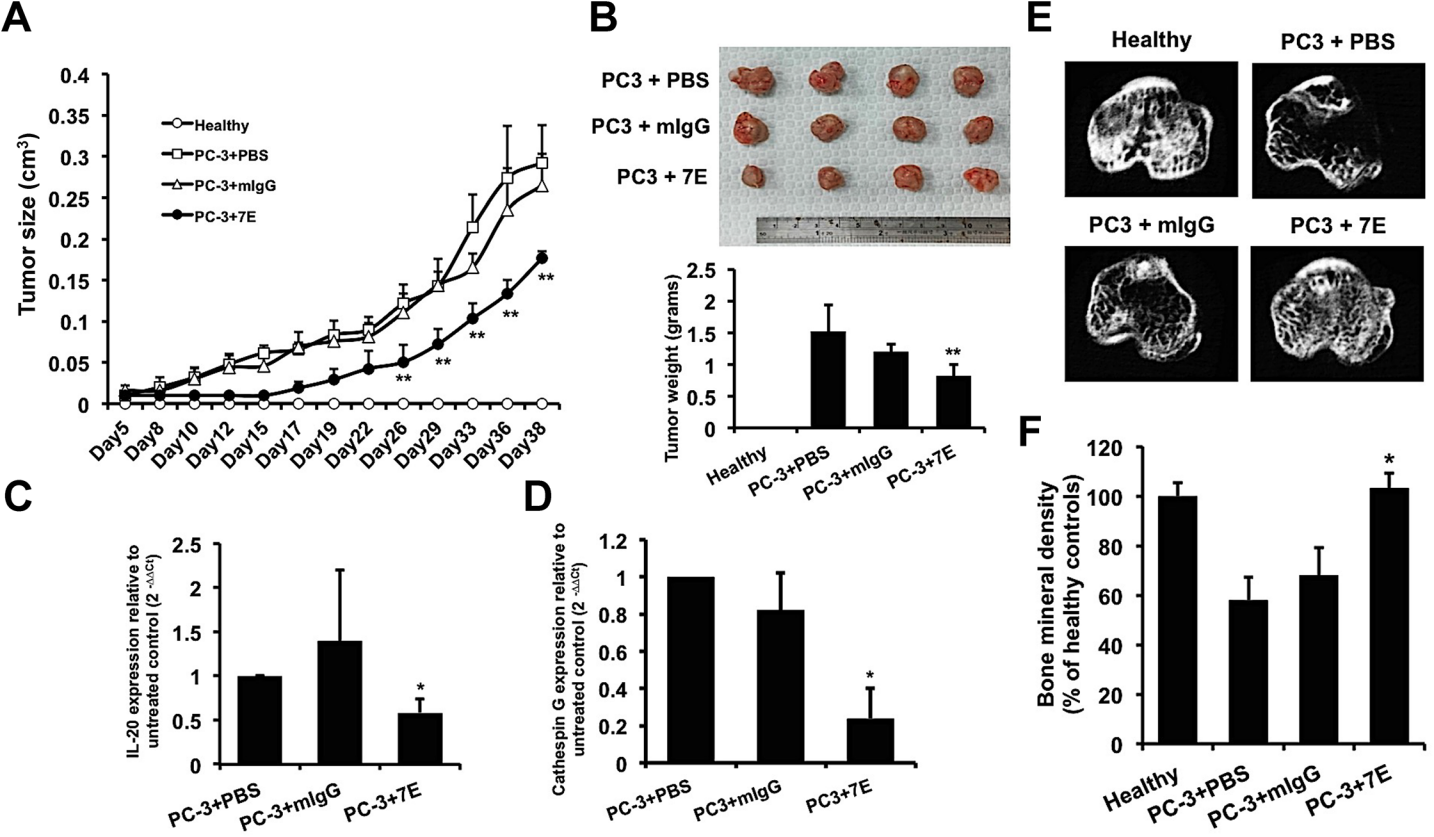
Anti-IL–20 mAb treatment suppressed tumor growth and protected against cancer-induced osteolysis *in vivo*. (A-D) PC–3 cells were injected (s.c.) into the mammary fat pads of SCID mice. One day later, the mice were injected (s.c.) with PBS, 7E (10 mg/kg), or mIgG (10 mg/kg; n = 4 per group) three times per week. (A) Tumor size was measured using a caliper. (B) Mice were killed 40 days after they had been injected with PC–3 cells, and their tumors were collected and weighed. (C-D) Tumor tissue samples from each group (n = 4) were isolated at the end of the experiment. The expression of IL–20 and cathepsin G in tumor tissue was analyzed using RT-qPCR with specific primers. *p < 0.05, **p < 0.01 versus mIgG controls. Data are means ± SD. Data are representative of 3 independent experiments with similar results. (E-F) 7E treatment protected against bone loss and lessened bone density decrement in intratibial PC-3-injected osteolytic mice. PC–3 cells (2×10^5^/100 μl) were slowly injected into the bone marrow cavity of the tibia. Mice were injected i.p. with PBS, 7E (10 mg/kg), or mIgG (10 mg/kg) (n = 5 in each group) three times per week. Healthy controls were not injected with cancer cells. (E) The tibial metaphysis was taken from healthy control mice and mice with PC-3-induced osteolysis. Representative micro-CT scans are shown for each group. (F) Tibial BMD was measured and the results expressed in percentages relative to healthy control values. *p < 0.05 versus mIgG controls. Data are shown as means ± SD. Data are representative of 3 independent experiments with similar results. BMD, bone mineral density; micro-CT, micro-computed tomography.

Based on our *in vitro* results, we further investigated the effects of 7E on prostate tumor growth *in vivo* in our mouse xenograft model. PC–3 cells were injected (s.c.) into SCID mice, which were then treated with PBS, mIgG (10 mg/kg, s.c.), or 7E (10 mg/kg, s.c.) injections 3 times per week, and tumor growth was measured for 40 days. There was less growth in the 7E-treated group than in the mIgG- and PBS-treated control groups (Fig 7A). After 40 days, the mice were killed and their tumors were weighed. Tumors in the 7E-treated group weighed less than in the mIgG- and PBS-treated groups (Fig 7B). Tumor tissue from each group was then isolated to extract RNA. RT-qPCR showed that IL–20 expression in the 7E-treated group was significantly lower than in the mIgG- and PBS-treated control groups (Fig 7C). In addition, cathepsin G expression was downregulated in the 7E-treated group (Fig 7D).

### 7E inhibited tumor-induced osteolysis in PC–3 prostate cancer xenografts

In the progression of prostate cancer, many patients eventually develop bone metastasis. Based on our *in vitro* data, we speculated that IL–20 might promote osteoclastogenesis by regulating sRANKL and cathepsin G expression in prostate cancer cells. Therefore, we wondered whether inhibiting IL–20 would reduce prostate cancer-induced osteolysis *in vivo*. PC–3 cells were injected into the bone marrow cavity of the tibias in SCID mice, which were then injected (i.p.) with PBS, mIgG, or 7E three times per week for the next 7 weeks. Micro-CT scans, used to examine the effect of 7E on prostate cancer-induced osteolysis, showed that PC–3 cancer-induced osteolysis was inhibited in the 7E-treated group compared with the mIgG- and PBS-treated groups (Fig 7E). BMD was significantly lower in the mIgG- and PBS-treated control groups than in the healthy control group; however, in the 7E-treated group, it was significantly higher than in the mIgG- and PBS-treated groups (Fig 7F). These data support our hypothesis that inhibiting IL–20 suppressed tumor-induced osteolysis *in vivo*.

## Discussion

The present study provides evidence that IL–20 is associated with the pathogenesis of prostate cancer. IL–20 and its receptors were all expressed in prostate tumor tissue specimens and prostate cancer cell lines (PC–3 and LNCaP). IL–20 affected prostate cancer cell migration and colony formation, and promoted tumor progression. IL–20 induced the expression of N-cadherin, STAT3, vimentin, fibronectin, RANKL, cathepsin G, and cathepsin K, all of which are involved in cancer cell migration, EMT, and tumor-induced osteolysis. Therefore, IL–20 directly affects tumor migration and progression, and indirectly affects these activities by inducing other mediators. We show that IL–20 is a local mediator in the microenvironment that affects prostate cancer cells.

IL–20 and its three receptors were expressed in our clinical specimens and in the PC–3 and LNCaP prostate cancer cell lines, which suggested both autocrine and paracrine effects of IL–20 on prostate tumors and contributions to the pathogenesis of prostate cancer. We did not show that IL–20 expression is associated with advanced tumor stages and patient survival because our patient data were obtained from only stage II and stage III prostate cancer, which is not late enough for analysis of advanced stage. More clinical follow-up analyses might provide additional evidence of the effects of IL–20 expression on clinical outcomes.

Although MTT assay analyses showed that IL–20 increased prostate cancer PC–3 cell proliferation, ANOVA showed no significant difference. We hypothesized that the endogenously expressed basal level of IL–20 is high enough to increase the proliferation of PC–3 cells. That anti-IL–20 mAb (7E) treatment dose-dependently inhibited the proliferation of PC–3 cells confirmed our hypothesis. In our mouse model, IL–20 was highly expressed in the PC–3 tumor cells. 7E’s inhibition of tumor growth was positively associated with the downregulated expression of IL–20 in the tumor mass. Therefore, these findings indicated that IL–20 promotes tumor development in prostate cancer *in vivo*.

The role of EMT in cancer cell invasion, migration, and metastasis is now well established for prostate cancer [33]. EMT allows cancer cells to detach from the extracellular matrix and provides a way for epithelium-derived tumors to survive as nonadherent cells and then enter the circulation. Other studies [30, 34] have shown that IL–20 upregulated invasion-related genes like MMP–9 and MMP–12, which degrade extracellular matrix in breast and bladder cancers. In the present study, we found that IL–20 regulated EMT-associated markers (E-cadherin, N-cadherin, vimentin, and fibronectin), affected prostate cell migration, and increased anchorage-independent growth in prostate cancer by inducing the phosphorylation of p38, ERK1/2, AKT, and NF-κB signals. Thus, we hypothesize that IL–20 produced by prostate cancer cells is important for inducing EMT in the primary tumor and then for promoting tumor cell migration and local invasion.

Cathepsins are critical mediators of metastasis across a range of tumors, including prostate cancer [35]. Cathepsin K found in the tumor-bone interface is responsible for the production of NTx, a marker of bone turnover, which is generated from the degradation of bone matrix collagen I [36]. Cathepsin G-mediated activation of MMP–9 at the tumor-bone interface promotes bone destruction in breast cancer [30, 37]. Cathepsin G acts as a chemoattractant for osteoclast precursors and increased RANKL-induced osteoclast differentiation [10, 38]. Our findings in this study showed that IL–20 promoted cathepsin K, cathepsin G, and RANKL expression, and induced sRANKL protein production in PC–3 cells. These data suggested that IL–20 is a regulator in the bone microenvironment of prostate cancer.

The dysregulation of osteoblast and osteoclast activity is responsible for the development of bone metastasis in prostate cancer. Other studies [39, 40] have reported that tumor cells express RANK and activate RANKL-RANK pathway. We previously [28] showed that IL–20 acted on osteoclast and induced RANK and cathepsin G, which modulates sRANKL production on osteoblasts. In the present study, we found that IL–20 directly induced the cleavage of RANKL from the surface membrane of prostate cancer cells by upregulating cathepsin G expression. RANKL stimulation induces the overexpression of several genes implicated in bone metastasis [41, 42]. These data provide potential mechanisms which explain that IL–20 not only triggers the entrance of prostate tumor cells into the bone microenvironment, but also increases prostate cancer-induced osteolysis by regulating the RANKL-RANK pathway and modulating osteoclastogenesis. 7E potently prevented bone loss in our PC-3-induced osteolytic model, which supports our hypothesis.

To clarify the biological function of IL–20 on PC–3 cells, we tested different dosage of IL–20 (50, 100, and 200 ng/ml), we found that 200 ng/ml of IL–20 is the optimal dose for *in vitro* assay. In our *in vivo* models, we tested three dosage of 7E (1, 5, and 10 mg/kg), the results showed that 10 mg/kg of 7E is the optimal dose for these tumor models.

IL–20 activates the type I (IL-20R1/IL- 20R2) and type II (IL-22R1/IL-20R2) receptor complexes, which are also activated by IL–24. Although IL–24 was reported [43–45] to be a tumor suppressor, the biological functions of IL–20 and IL–24 are different. Whether IL–24 is involved in the pathogenesis of prostate cancer awaits further investigation. Moreover, we found that IL-20R1 was highly expressed in most of the patients with prostate cancer enrolled in this study. Whether IL-20R1 is a biomarker for predicting bone metastasis in prostate cancer needs additional investigation.

In conclusion, our study demonstrates that IL–20 has an autocrine effect and provides a microenvironment that affects tumor progression and prostate cancer-induced osteolysis. Antagonizing IL–20 might have therapeutic potential in prostate cancer.
